# EHMTI-0064. The prevalence of cluster headache in the elderly is higher in women than in men

**DOI:** 10.1186/1129-2377-15-S1-C36

**Published:** 2014-09-18

**Authors:** C Lisotto, F Mainardi, F Maggioni, G Zanchin

**Affiliations:** 1 Department of Neurosciences, University-Hospital of Padua, Padova, Italy

## Introduction

Cluster headache (CH) is considered a disorder of young men, which predominantly begins at age 20-to-40 years.

## Aims

We evaluated the gender distribution in patients with CH aged 65 years and older.

## Methods

For the last 15 years we have observed 261 patients suffering from CH. Out of these cases, 43 patients (16.5% of the whole population) were over 65 years.

## Results

In this group of elderly patients, 24 were females and 19 were males. We diagnosed 4 patients with CH (only one bout, according to the International Classification of Headache Disorder), 25 with episodic CH, and 14 with chronic CH. The onset occurred in ages 35-44 years for 21.4% of cases, in ages 45-54 for 16.7%, in ages 55-64 for 28.6% and after the age of 65 years for 33.3%. Notably, in the latter subgroup, the females significantly prevailed, accounting for 78.6% of cases. Out of the patients aged over 65 years the prevalence of chronic CH was remarkably higher (25.6%) than in previous ages (9.5%).

## Conclusions

The increasing number of elderly patients with CH could be related to an inadequate recognition of this headache disorder, which has been believed for a long time to affect mainly young subjects. Apparently peculiar to female distribution, an increased frequency of CH appears to occur in middle-age and elderly patients. To our knowledge, we report the patient with the oldest age at onset (a 93-year-old woman) and the largest case series of CH elderly patients published in the literature to date.

No conflict of interest.

